# Harmful uses of patentable neurotechnology: a new regulatory approach: Proposing a framework safeguarding human rights and social responsibility for patented applications of neuroscience

**DOI:** 10.1038/s44319-024-00129-2

**Published:** 2024-04-05

**Authors:** R Roland Nadler, Tade M Spranger, Ari Rotenberg, Tanya A Barretto, Julia Hansmann, Anna Hemmer, Zelma HT Kiss, John DW Madden, Michael J Strong, Judy Illes

**Affiliations:** 1 Peter A. Allard School of Law, University of British Columbia, Vancouver, Canada; 2 Neuroethics Canada, Division of Neurology, Faculty of Medicine, University of British Columbia, Vancouver, Canada; 3 Centre for the Law of Life Sciences, Rechts- und Staatswissenschaftliche Fakultät, Universität Bonn, Bonn, Germany; 4 Hotchkiss Brain Institute, Cumming School of Medicine, University of Calgary, Calgary, Canada; 5 Department of Electrical and Computer Engineering, University of British Columbia, Vancouver, Canada; 6 Department of Clinical Neurological Sciences, Schulich School of Medicine & Dentistry, Western University, London, Canada

**Keywords:** Economics, Law & Politics, Neuroscience

## Abstract

The increasing ability of neuroscience to analyse and modulate human brain functions calls for a new regulatory approach to identify and deal with potential harmful applications in the early stages of development.

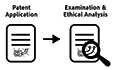

The brain as executor of the mind shapes perception, identity, personality and expression. By interacting with neural activity, neurotechnology can thus reveal and even modify an individual’s mental abilities and experience. With approximately 1 in 3 people worldwide expected to suffer from a neurologic disorder at some point in their lifetime (Feigin et al, 2020), developing neurotechnology to treat diseases and improve quality of life is a global economic and public health priority. Yet, healthcare is only one front of research and development. Industries such as marketing and resource management have also adopted neurotechnology to evolve new practice standards (Greenberg et al, 2021). On the technological horizon loom sophisticated brain-computer interfaces with functions going well beyond the therapeutic. Alongside the potential benefits of private companies developing technologies that can intervene with brain functions, exceptional ethical, legal and social risks arise.

On the technological horizon loom sophisticated brain-computer interfaces with functions going well beyond the therapeutic.

Addressing socially harmful uses of neurotechnology requires a full spectrum of regulatory institutions acting in cooperation. Typically, however, these regulators must wait for concrete harms to arise before they form an understanding of the problem and act accordingly. In other words, governments are principally equipped to address harmful impacts of neurotechnology in a reactive manner, rather than a proactive one. Noting the substantial risks afoot, we consider this watchful-waiting approach insufficient. More active vigilance is required. Pre-market review imposed by health authorities can preemptively limit the potential harm of medical devices, but medical regulators can hardly meet all emerging needs. In this article, we propose a coordinated and distributed regulatory strategy designed to mitigate socially harmful uses of neurotechnology.

… governments are principally equipped to address harmful impacts of neurotechnology in a reactive manner, rather than a proactive one.

## Background

For many years, experts in neuroscience have called for meaningful engagement between regulators and innovators (Garden et al, 2019). While widespread cooperation has proved an elusive goal, some innovators have opted for self-regulation as a compromise, advertising stringent user privacy and safety commitments. Examples include InteraXon, which markets affordable neurotechnology directly to consumers, and Nielsen, which uses insights from neuroimaging to optimize consumer engagement with media (Ienca et al, 2018). However, these entities stand as exceptions; rarely do plans for socially responsible product development enjoy vetting from experts in positions of public accountability. Meanwhile, mushrooming patent filings and market capital valuations reveal that the neurotechnology sector is growing rapidly (Hain et al, 2023). As new entrants surge to the marketplace, public oversight will offer a more reliable avenue for promoting responsible practices.

The important task of designing regulatory frameworks for commercial neurotechnology will need to address several challenges. On the one hand, there is difficulty in balancing pre- and post-market regulatory strategies. It would be reckless to accept an “innovate first, regulate later” mentality even if the eventual regulation is robust and effective. On the other hand, no system can prospectively enumerate every possible problem that might arise as novel neurotechnologies reach consumers. Moreover, categorically subjecting neurotechnology to multiple onerous pre-clearance processes would stifle innovation and entrepreneurship. We commit to no such extremes. Instead, we envision regulators, prompted by advance warnings from an appropriately situated patent system, engaging with innovators and developing plans to manage the most realistic, foreseeable risks.

The key feature of the proposed regulatory strategy lies in leveraging existing patent systems to monitor and flag potentially harmful technologies. Since almost all neurotechnologies will initially pass through a patent examiner’s hands, this is where society ought to situate infrastructure for assessing risks and issuing early warnings. We characterize this proposed approach as distributed because it allocates further responsibility for acting on early warnings to a wide variety of institutions. These institutions include familiar agencies such as health or consumer-protection regulators, but also human-rights bodies, legislatures, law enforcement, expert councils, and more.

**Figure 1:**
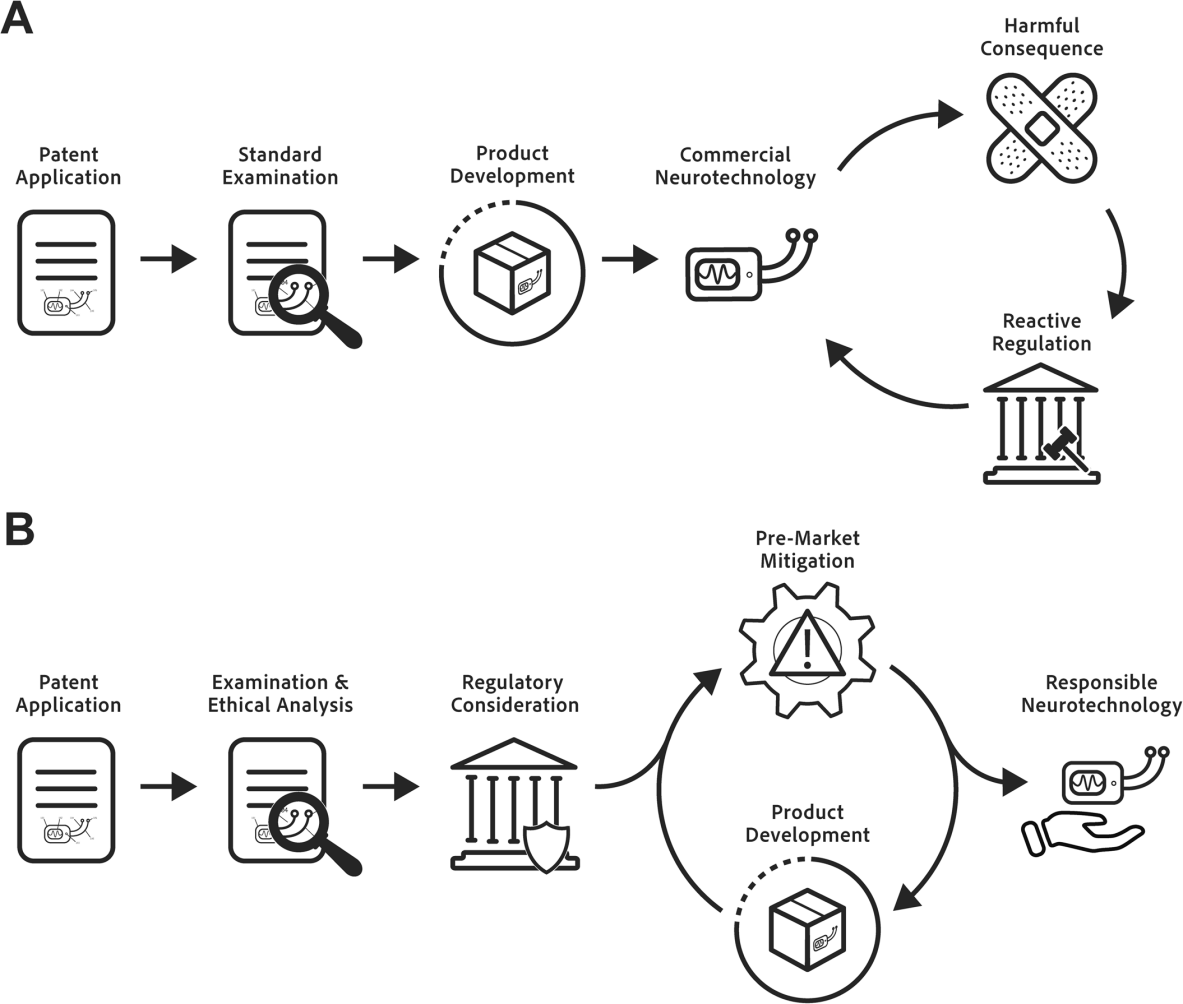
Current and proposed regulatory strategies for neurotechnology patents. (**A**) Current reactive regulatory approaches act on risks after harms are realized. While not shown, medical products subject to pre-market approval still undergo that process. (**B**) The proposed anticipatory regulatory strategy, in which patent offices may prompt early regulatory engagement. Note that successive rounds of interaction between developer and regulator may also take place post-market.

The paradigm we set out here entails novel but straightforward forms of coordination between patent systems and regulatory bodies. This would allow them to accomplish together what they have been unable to do separately: scan for potential harmful uses of neurotechnology, identify areas of concern, and apply appropriate regulatory tools early to curb incipient risks (Fig. 1). This strategy will better position government agencies to anticipate and avert the social and human rights challenges posed by burgeoning neurotechnologies. It also creates an opportunity to foster cooperative and communicative relationships between regulatory bodies and private innovators.

## Patent systems as monitors

We envision a key role for patent offices in the proposed regulatory approach for neurotechnology. Specifically, they would evaluate neurotechnology patents for potential social harms at the time of examination, and either directly notify relevant regulatory agencies or simply make evaluation results broadly available at the time of publication for granted patents. Examples of such harms might include undue infringements on privacy, autonomy or mental integrity; extraordinary health or safety risks for devices exempt from medical regulation; or latently anti-consumer product designs that leave end-users vulnerable to unilateral alterations of device functionality.

Since almost all neurotechnologies will initially pass through a patent examiner’s hands, this is where society ought to situate infrastructure for assessing risks and issuing early warnings.

There are several reasons why patent systems are ideally positioned to detect emerging neurotechnologies that require regulatory attention. First, patent publications are the earliest form of communication between innovators and the public regarding products under development. In some cases, patenting may be the public’s only glimpse at a developing technology until it debuts on the market. Due to the enablement requirement in patent law, documents are highly detailed; they often delineate examples of the invention’s operation within a foreseeable context.

Further, the widespread adoption of first-to-file systems by intellectual property offices has incentivized inventors to apply for patents as early as possible. This tends to maximize the time between patent publication and product release, which could work to the advantage of regulators—as long as key information circulates to them. Although inventors have no legal obligation to seek intellectual property protections, the ubiquity and inherent value of patents makes it likely that most emerging neurotechnologies, including ethically risky ones, will interface with the patent system at some time in their development. One might visualize the patent system as a long and transparent funnel; developing technologies enter long before they exit, and so it becomes possible to scrutinize all the technologies as they pass through the funnel’s narrowest point. This narrow point is where ethical analysis should begin—not in the form of policymaking but simply in the form of issue-spotting.

At least under the umbrella of the European Patent Convention (EPC), which as a multilateral treaty shapes the patent law of almost 40 countries, the task we are envisioning for patent offices can be carried out straightforwardly. Patent examiners at the European Patent Office (EPO) already routinely check for conformance with Art. 53(a) EPC. (EPO, 2023), which denies patentability to inventions whose commercial exploitation would be contrary to “ordre public” (i.e., public policy or morality). European patent examiners are thus already legally obligated to assess whether a given patent raises concerns about potential socially harmful uses as part of the routine workflow of examination without further training.

European patent examiners are thus already legally obligated to assess whether a given patent raises concerns about potential socially harmful uses…

Article 53(a) EPC alone is not, however, sufficient to deal with potential socially harmful uses of neuroscientific inventions; patentability remains intact so long as even one unobjectionable commercial use is conceivable (Spranger, 2023). As the EPO has stated: “This provision is likely to be invoked only in rare and extreme cases … [where] the public in general would regard the invention as so abhorrent that the grant of patent rights would be inconceivable…. The mere possibility of abuse of an invention is not sufficient … ” (EPO, 2023).

A more sustainable and less categorical approach would couple the existing obligation to carry out an ethical examination with a mechanism to flag and forward relevant patents to other institutions with the necessary expertise and authority to regulate for safety. If an examiner can plausibly foresee a scenario where the mature version of the proposed technology causes injury, facilitates unethical conduct, or undermines human rights, it should be noted accordingly. This would then trigger a procedure to report, or passively make the notation visible, to the relevant organizations or authorities. While the EPC itself can only be amended jointly by all member states (Vienna Convention, 1980, Art. 40.2), such a reporting obligation can be implemented via the Guidelines for Examinations, which are revised annually by the European Patent Office (EPO, 2024). Furthermore, it would neither lead to higher costs nor overburden patent examiners’ resources and expertise, as examiners already operate under Art. 53 (a) EPC.

Depending on the institutional characteristics of a given patent office, the details of implementation for this monitoring function may need to vary considerably. In some systems outside Europe, it may be unrealistic to assign monitoring responsibilities to patent examiners themselves. In this case, specially trained personnel or external experts could be brought on for the role. We recognize that patent systems are underfunded, and so it may not be reasonable to ask examiners to take on a new task even with proper training and additional compensation. Nonetheless, we urge policymakers to recognize that addressing socially harmful uses of neurotechnology is a priority worth investing in, and that a mindset of austerity on this issue may eventually lead to social harms far more expensive to redress than they would have been to prevent. Practical realities will dictate different specifics for this monitoring procedure within each patent system; what matters is that risky neurotechnology patents exit any given system properly flagged for the attention of more suitable regulators.

It is reasonable to ask: if examiners designate a neurotechnology as risky in this way, why should it be patentable at all? In general, patent law is presumptively ethically neutral (Recital 14) but major jurisdictions including Europe and the USA do make limited exceptions to this principle, via Art. 53 (a) EPC or by forbidding patenting of weaponry such as landmines (EPO, 2023) and atomic weapons (42 USC § 2181, 2018). All the same, we do not recommend adding risky neurotechnologies to this short list of categorical bans. Doing so would be disproportionate, overstepping the understood purpose of patent law. Its limitations and principles of neutrality exist for good reason. Denying patentability altogether would also impose novel restrictions on commercial markets, quashing new and valuable fronts of innovation.

By contrast, a simple monitoring role would only modestly extend the current functioning of patent systems, if at all. While patent examiners should not be tasked with bottom-line technology regulation, they are as capable as the public servants staffing other regulatory agencies of foreseeing ethical issues stemming from novel technologies. Foresight of risks gives rise to important opportunities for analysis, preparation, mitigation and prevention.

## Downstream agencies as regulators

With responsibility for early detection of potential neurotechnological harms delegated as far upstream as possible, the work of preventing and redressing those harms can be taken up by appropriately specialized actors. The nature and structure of regulatory agencies varies widely; it is impossible to comprehensively specify the procedures that each institution would apply upon receiving advance warnings. Instead, we offer in this section some representative conceptual examples grouped across different categories.

While health and drug regulators cannot manage neurotechnological issues unaided, they will always retain a prominent role. Many issues raised by emergent neurotechnologies would fall squarely within the respective jurisdictions of bodies such as Health Canada, the US Food and Drug Administration (FDA), the European Medicines Agency, the Medical and Healthcare products Regulatory Agency in the UK, or the Australia Therapeutic Goods Administration. Overall, the proposed approach would entail minimal changes to this regulatory sector, largely because medical device manufacturers are already accustomed to making early contact with the relevant agencies to proactively navigate the approval process.

Advance warnings from the patent system about risky neurotechnology would considerably alter the dynamics of privacy and consumer-protection regulation. Privacy concerns are likely to represent a large proportion of foreseeable risks, since almost all relevant technologies will collect and store users’ brain data. Accordingly, both purpose-built entities such as the European Data Protection Supervisor or the Office of the Australian Information Commissioner, as well as larger institutions with privacy-protection responsibilities such as the US Federal Trade Commission (FTC), would be well-positioned to handle many concerns. Instead of waiting for breaches of privacy laws to arise, these agencies could develop robust guidelines and invite holders of flagged patents to work together on plans for compliance before products launch.

Patented neurotechnology may also pose risks best addressed through consumer-protection infrastructure. For example, companies providing brain-computer interface (BCI) technologies might find it profitable to unilaterally alter or limit the functionality of their products in a manner similar to digital rights management regimes practiced by dominant media platforms today. Such interventions not only limit users’ control over the functioning of their own device-integrated brains, but also assert a form of corporate control in the same sphere. An agency like the Australian Competition and Consumer Commission or, again, the US FTC would have the appropriate expertise to address such practices. To the extent that anti-consumer designs or practices become embedded in neurotechnology firms wielding excessive market power, there may be an eventual role for competition regulators in this space as well.

We anticipate that governmental human rights tribunals or commissions—for example, national anti-discrimination authorities or the Council of Europe and its European Court of Human Rights—will eventually find themselves addressing complaints that arise from neurotechnology. Since some of these institutions function as courts, they will tend to address issues reactively rather than proactively via adjudication and remedies. However, human rights bodies need not be limited strictly to backward-looking measures. With the benefit of information received from patent systems, these tribunals and commissions could analyze specific neurotechnology-linked rights concerns and educate the public accordingly. Where a neurotechnology poses a specific risk to human rights that cannot be eliminated by proactive regulation—for example, harms to mental integrity stemming from device malfunction or security flaws—a human rights tribunal might work with the patent holder to establish an orderly and efficient claims process for individuals whose rights may later become infringed.

In common-law systems, the office of the attorney general enforces existing laws by taking alleged violators to court. Whether using general consumer-protection provisions or novel statutory frameworks more purpose-built for neurotechnology regulation, attorneys general will be able to hold companies accountable if they break the law. Unlike regulatory agencies, these civil law enforcement actors have little capacity to proactively assist patent holders in developing legal compliance strategies. However, early warnings from the patent system would still help attorney general offices know where to eventually look for potential breaches of the law.

Legislative bodies such as federal parliaments and subnational state houses or general assemblies are also in a good position to act on early warnings. They can do so principally by enacting laws, following the example set in previous decades by novel statutory frameworks governing, for example, genetically modified organisms or stem-cell research. However, legislatures also have considerable power to find facts, a power they exercise through inquiries, investigations, hearings, reports and more. A well-functioning legislature is an integral part of any distributed regulatory approach to social problems because it is especially well-positioned to coordinate among other actors while at the same time remaining answerable to voters, ensuring democratic accountability.

Some institutions with important regulatory functions may be thought of as quasi-governmental—in this case, colleges of physicians, professional engineering societies, law societies or bar associations, and other professional societies at both federal and subnational levels—that can contribute to a strategy for neurotechnology regulation as well. Early warnings about risky patents would enable them to make appropriate preparations. For example, colleges of physicians could contemplate and craft professional rules governing ethical uses of physician-aided neuromodulation. Indeed, they may prove better suited to this task than generalist legislatures. Lawmakers or political leaders could even direct the college of physicians in their jurisdiction to draw up and promulgate suitable rules for doctors within its purview.

As we canvass quasi-governmental regulatory institutions, special attention is warranted to research oversight bodies. Whether in the form of state-run clinical trial overseers, university research ethics boards, or funding bodies such as the Canadian Institutes of Health Research or the US National Science Foundation, these entities also fit into the regulatory strategy as we envision it. These types of actors would be especially able to make use of early warnings about risky neurotechnology patents because the path from patent to realized technology often runs through human-subjects research. Finally, and furthest attenuated from the actual government, there are civil society groups, non-government organizations (NGOs), and advocacy organizations that can effectuate meaningful regulatory force on technologies even without the backing of law.

Two important points emerge from these examples. First, effective regulation of neurotechnology for social responsibility will require the involvement of a broad cross-section of the administrative state, plus other non-governmental actors. Second, the proposed approach generates valuable opportunities for private industry and public officials to work collaboratively towards the goal of delivering socially responsible neurotechnology.

… the proposed approach generates valuable opportunities for private industry and public officials to work collaboratively towards the goal of delivering socially responsible neurotechnology.

## Case example

By way of example, consider the case of the dual use of neurotechnology. Suppose a delivery service seeks to implement an EEG headset system to monitor drivers’ attentiveness and neurologic health (Dolezalek et al, 2021; Guo et al, 2017). Whereas a public safety rationale—that is, prevention of accidents—is plausible in this case, the device could equally surveil driver activity in a manner that deters breaks and incentivizes overwork. In other words, the neurotechnology is not inherently harmful, but its foreseeable uses verge into territory that is exploitative and may threaten human privacy and dignity.

How does the proposed approach address the dual-use problem in this example? The answer begins with an early warning about the EEG device communicated from the patent office to appropriate institutions—here, the National Labor Relations Board, as well as the US FTC. Advance notice is what empowers these agencies to collaboratively formulate plans for addressing the issue. These plans might involve certain design specifications from the manufacturer before marketing begins, thereby rendering it impossible to use the EEG headset in ways that would amount to an unfair labor practice. Failing that, officials might develop technology-specific protocols for swiftly detecting and addressing any unfair labor practices linked to the device as they arise.

Instead of remaining mired in an old-fashioned regulatory paradigm where government and industry maintain an adversarial relationship and spar over enforcement actions, vested stakeholders can work towards a more cooperative model of governance championed by forward-thinking scholars of the administrative state (Freeman, 1997). The proposed approach opens up these avenues for improvement thanks to the early notifications furnished by the patent system.

## Conclusion

A broad constellation of government agencies, public officials, and quasi-governmental organizations will be needed to help meet the challenge of minimizing harmful uses of neurotechnology for society. We reiterate our view that the status quo is untenable: simply leaving the regulatory state to respond to problems with neurotechnology as they arise will invite avoidable harm. The distributed approach that we have proposed facilitates effective regulatory intervention both pre-market and post-market. A patent flagged by examiners as potentially concerning would be addressed prospectively, for example, via more stringent review from a health regulator or additional guardrails imposed by a clinical trial oversight body. In some cases, it may still be necessary to address issues retrospectively, for example, via an enforcement action by a consumer-protection agency or a state attorney general. The distributed approach also enables jurisdictions to match each type of potential problem to the best-suited actor. Different institutions have their own distinctive expertise, powers and cultures. As the potential risks of the neurotechnological future will take many different forms, so too must the policy tools for addressing them be accordingly diverse.

… simply leaving the regulatory state to respond to problems with neurotechnology as they arise will invite avoidable harm.

### Supplementary information

Peer Review File
