# Supplementary material for: Harmful uses of patentable neurotechnology: a new regulatory approach: Proposing a framework safeguarding human rights and social responsibility for patented applications of neuroscience
Source: EMBO Rep. 2024 Apr 5;25(5):2156–61. doi: 10.1038/s44319-024-00129-2 (PMC11094056; doi:10.1038/s44319-024-00129-2)
Supplement: Supplementary file 1 — Peer Review File [file 44319_2024_129_MOESM1_ESM.pdf]

EMBO reports

Harmful Uses of Patentable Neurotechnology: A New Regulatory Approach

R. Roland Nadler, Tade Spranger, Ari Rotenberg, Tanya Barretto, Julia Hansmann, Anna Hemmer, Zelma Kiss, John Madden, Michael Strong, and Judy Illes

Corresponding author(s): R. Roland Nadler (nadler@student.ubc.ca) , Tade Spranger (tadespranger@web.de)

Review Timeline:

Submission Date:	17th Feb 24
Accepted:	20th Mar 24

Editor: Holger Breithaupt

Transaction Report: This article was editorially reviewed.
